# Supplementary material for: The role of hesitancy and infrastructure in the equity and efficiency of COVID-19 vaccine administration
Source: PLoS One. 2024 Jun 14;19(6):e0304416. doi: 10.1371/journal.pone.0304416 (PMC11178164; doi:10.1371/journal.pone.0304416)
Supplement: Attachment — Submitted filename: Response to Reviewers.pdf [file pone.0304416.s002.pdf]

We would like to thank all the reviewers and the editor for taking the time to review and provide comments on our manuscript. We appreciate that you see intellectual merit in our work and are grateful for the opportunity to strengthen the paper by incorporating your feedback.

Below, we have itemized the reviewer's comments and correspondingly provided a description of the changes that were made to the manuscript incorporating the feedback. Where appropriate, we have included an excerpt from the revised text.

Reviewer 1 Comments:

1. *there is some repetitiveness in the introduction and literature review. The research questions are stated at the end of the introduction, so the aims don't need to be mentioned again in the first paragraph of the literature review, and if they do, then they have not been explained well enough.*

   Thank you for mentioning this. We have trimmed down the literature review so that it immediately addresses the relevant topics and studies without repeating the aims of our study. The updated text is included below.

   > [Line 73]
   > **2.2 Vaccine Administration Efficiency**
   > Much like with equity, there are different lenses through which scientists have studied efficiency of vaccine administration.
   >
   > [Line 92]
   > **2.3 Vaccine Hesitancy**
   > Lastly, utilize the hesitancy rates that were observed during the COVID-19 pandemic to discern whether states' VEAPs aligned with their hesitancy rates.

2. *lines 196-250 has question marks for the figure numbers which need removing.*

   The figure numbers have been corrected in the updated manuscript.

3. *can you explain more fully why the 5 states included were chosen? Was this random or due to data availability or other criteria?*

   The first paragraph of Section 3 was changed to more explitely state that the 5 states were chosen randomly and were kept because the resulting sample had diverse population sizes, densities, political representation, COVID-19 dynamics, and their entire vaccine eligibility timeline published. The updated text is included below.

   > [Line 102]
   > **3 Methods**
   > To address the research objectives stated above, we examined vaccine allocation and administration in five US states: California, Massachusetts, Mississippi, New Mexico, and Ohio. These states were selected randomly, and were used in the final analysis because they met two criteria. First, this sample of states has different population densities [39], locations, times of first COVID-19 peak [9], and political leadership in the

state legislature [28] in early 2020, shown in Table 1. These attributes make the sample robust to biases associated with geography, political leadership, or size. Second, the selected states all had the entire vaccine-eligibility timeline published on public websites, which was not the case for all US states. Each state had a unique platform for communicating eligibility information and timing. Many of these platforms only showed the most up-to-date information, not the entire eligibility timeline.

4. *please explain in the limitations section that the sources of data used were very different and therefore not generalisable e.g. media was used for one*

   Section 5.1 was updated to articulate the limitations of the study more clearly (i.e., small sample size and decentralized data collection) and to clarify how using separate sources to get data from each state could impact the generalizability of the results. The updated text is included below.

[Line 306]
5.1 Limitations
Two primary limitations of this work include (1) the use of a small sample of states and (2) the use of decentralized data sources to construct VEAPs and eligible population profiles for each state. This study only examines the efficiency and equity of vaccine distribution in 5 of the 50 US states. This small sample size was chosen partially due to the lack of available information on states' eligibility criteria and also the decentralized nature of this data. Thus, we are careful about drawing broad conclusions about the overall success of states' vaccine administration campaigns. However, considering how consistent the discrepancy between vaccine supply and true demand were in each state, and the variety of states included in this study, it is unlikely that the small sample set of states are hindering the generalizability of these results. Since, during our initial selection of states, none of the 50 states stood out as having slow eligibility plans, that would more closely match federal vaccine supply, our results would be unlikely to change by examining additional states.

As previously mentioned, this study collected data from several public, non-government sources to calculate the results. Thus, the accuracy of our results is dependent on the accuracy of the data sources used and our assumptions about constant population size and uniform dispersion of hesitancy. Additionally, the use of decentralized sources limits the comparability of results between states. However, the sources used to find the total eligible populations, sizes of sub-populations, vaccines allocated, and vaccines administered were all collected from the same sources for each of the five states, which are the primary data that the results rely on. Thus, the accuracy and generalizability of results shown in this study is strictly dependent on the accuracy of the individual sources. This study's reliance on decentralized, non-government sources points to the need for improved data collection methods during future vaccine administration campaigns so that research reflecting on the success of the campaigns can be as accurate as possible.

5. in the future work section, you mention the use of models - do any of these already exist?

   Yes; Section 6.1 was updated to reference established research and models. Additionally, we further articulated how future work could expand upon these. An excerpt of the updated manuscript is included below.

6.1 Future Work

There are a variety of mathematical models and simulation tools that could be developed to inform the design of such an adaptive vaccine allocation system. In this study, we have highlighted key parameters that should be incorporated into this system including vaccine hesitancy, sub-population sizes, total eligible demand, and vulnerability. There is a need to utilize these concepts to develop models of a nation-wide vaccine administration campaign with the goal of maximizing efficiency, equity, and safety in real time. Such models can build on vaccine allocation models such as those developed by Balcik et al. [3] and Dastgonshade et al. [13]. These models could then be adapted to incorporate real data from multiple states to help orchestrate future mass-vaccination campaigns.

Reviewer 2 Comments:

- *This study reviewed five states; VEAPs, federal vaccine allocations, vaccine administrations, and vaccine hesitancy to assess the equity of vaccine access and vaccine administration efficiency that manifested during the COVID-19 vaccine campaign. The manuscript has many strengths, including the introduction and literature review. The information presented was clear and concise. The analysis was also very strong, with a thorough description of how equity, efficiency, and hesitance was assessed.*

  We appreciate your thorough review and feedback.

- *There were two grammatical errors/typos (line 184, line 207).*

  Both of these typos are fixed in updated manuscript.

  > [Line 184]
  > Data on the total number of people who received at least one dose of the vaccine was obtained from USA Facts [40].
  >
  > [Line 206]
  > The similar trajectory of the traces in Fig 1b suggests that the federal government allocated vaccines to states proportionally to their population throughout the campaign [10].

- *The manuscript implications section was especially interesting, as it provided suggestions for ensuring equitable and efficient distribution of vaccines or medical resources during health crises.*

  Thank you, we hope this work can be used to inform future vaccination campaigns and inspire additional work on allocation models that maximize equity of vaccine access.

In addition to the edits made to address the comments above, we have made changes to fix typos, clarify wording, and create consistency throughout the document. A copy of the manuscript with tracked changes (in red) is included in the resubmission, along with a clean revised copy that follows the Latex template provided in the revision instructions.
